# US biofuel production and policy: implications for land use changes in Malaysia and Indonesia

**DOI:** 10.1186/s13068-020-1650-1

**Published:** 2020-01-18

**Authors:** Farzad Taheripour, Wallace E. Tyner

**Affiliations:** Department of Agricultural Economics, Purdue University, West Lafayette, USA

**Keywords:** Induced land use change, Peatland conversion, US biofuel policy and impacts, Malaysia and Indonesia, Land use emissions

## Abstract

**Background:**

It has been argued that the US biofuel policy is responsible for the land use changes in Malaysia and Indonesia (M&I). In this paper, following a short literature review that highlights the relevant topics and issues, we develop analytical and numerical analyses to evaluate the extent to which production of biofuels in the US alters land use in M&I. The analytical analyses make it clear that market-mediated responses may generate some land use change in M&I due to biofuel production in the US. These analyses highlight the role of substitution among vegetable oils in linking these economies in markets for vegetable oils. To numerically quantify these effects, we modified and used a well-known Computable General Equilibrium model (CGE), GTAP-BIO. We conducted some sensitivity tests as well.

**Results:**

According to the simulation results obtained from two base case scenarios for corn ethanol and soy biodiesel, we find that producing 15 BGs of corn ethanol and 2 BGs gallons of soy biodiesel together could potentially increase area of cropland in M&I by 59.6 thousand hectares. That is less than 0.5% of the cropland expansion in M&I for the time period of 2000–2016, when biofuel production increased in the US. The original GTAP-BIO model parameters including the regional substitution rates among vegetable oils were used for the base case scenarios. The estimated induced land use change (ILUC) emissions values for corn ethanol and soy biodiesel are about 12.3 g CO_2_e MJ^−1^, 17.5 g CO_2_e MJ^−1^ for the base case scenarios. The share of M&I in the estimated ILUC emissions value for corn ethanol is 10.9%. The corresponding figure for soy biodiesel is much higher, 78%. The estimated ILUC emissions value for soy biodiesel is sensitive with respect to the changes in the regional rates of substitution elasticity among vegetable oils. That is not the case for corn ethanol. When we replaced the original substitution elasticities of the base case, which are very large (i.e., 5 or 10) for many regions, with a small and uniform rate of substitution (i.e., 0.5) across the world, the ILUC emissions value for soy biodiesel drops from 17.5 g CO_2_e MJ^−1^ to 10.16 g CO_2_e MJ^−1^. When we applied larger substitution elasticities among vegetable oils, the estimated ILUC emissions value for soy biodiesel converged towards the base case results. This suggests that, other factors being equal, the base case substitution elasticities provide the largest possible ILUC emissions value for soy biodiesel. Finally, our analyses clearly indicate that those analyses that limit their modeling framework to only palm and soy oil and ignore other types of vegetable oils and fats provide misleading information and exaggerate about the land use implications of the US biofuels for M&I.

**Conclusion:**

(1) Production of biofuels in the US generates some land use effects in M&I due to market-mediated responses, in particular through the links between markets for vegetable oils. These effects are minor compared to the magnitude of land use change in M&I. However, because of the high carbon intensity of the peatland the emissions fraction of M&I is larger, in particular for soy biodiesel. (2) The GTAP-BIO model implemented a set of regional substitution elasticities among vegetable oils that, other factors being equal, provides the largest possible ILUC emissions value for soy biodiesel. (3) With a larger substitution elasticity among all types of vegetable oils and animal fats in the US, less land use changes occur in M&I. That is due to the fact that a larger substitution elasticity among vegetable oils in the US, diverts a larger portion of the additional demand for soy oil to non-palm vegetable oils and animal fats that are produced either in the US or regions other than M&I. (4) Those analyses that limit their modeling framework to only palm and soy oils and ignore other types of vegetable oils and fats provide misleading information and exaggerate about the land use implications of the US biofuels for M&I.

## Background

### Literature review and major contributions

The land use change effects of biofuel production and policy has been examined frequently during the past decades. The early projections of these effects raised major concerns regarding the magnitude of the land use change emissions that biofuel production may generate at the global scale [1–3]. In the absence of actual observations, the early projections were basically obtained from hypothetical ex ante analyses [4]. For example, about one decade ago, Searchinger et al. [3] argued that producing corn ethanol in the US will generate 107 grams of CO_2_ equivalent per mega Joule (g CO_2_e/MJ) emissions due to direct and indirect land use changes that will happen across the world. These authors used an early version of a partial equilibrium model developed at the Food and Agricultural Policy Research Institute (FAPRI) to calculate this figure. With this projection, Searchinger et al. [3] argued that production of biofuels could generate more emissions than the traditional fossil fuels. This argument prompted several publications that have shown Searchinger et al. [3] overestimated induced land use change (ILUC) emissions due to biofuels. For example, in a seminal work, Hertel et al. [5] argued that Searchinger et al. [3] ignored several important factors such as market-mediated responses, resource constraints, and yield improvements in their evaluation for ILUC emissions. These authors used a Computable General Equilibrium (CGE) model that takes into account these important factors and projected a significantly lower ILUC value for the US corn ethanol, 27 g CO_2_e/MJ. For this evaluation, Hertel et al. [5] used the GTAP-BIO model. Following these initial estimates, many papers have estimated induced land use emissions for alternative biofuels that are produced across the world.

**Fig. 1:**
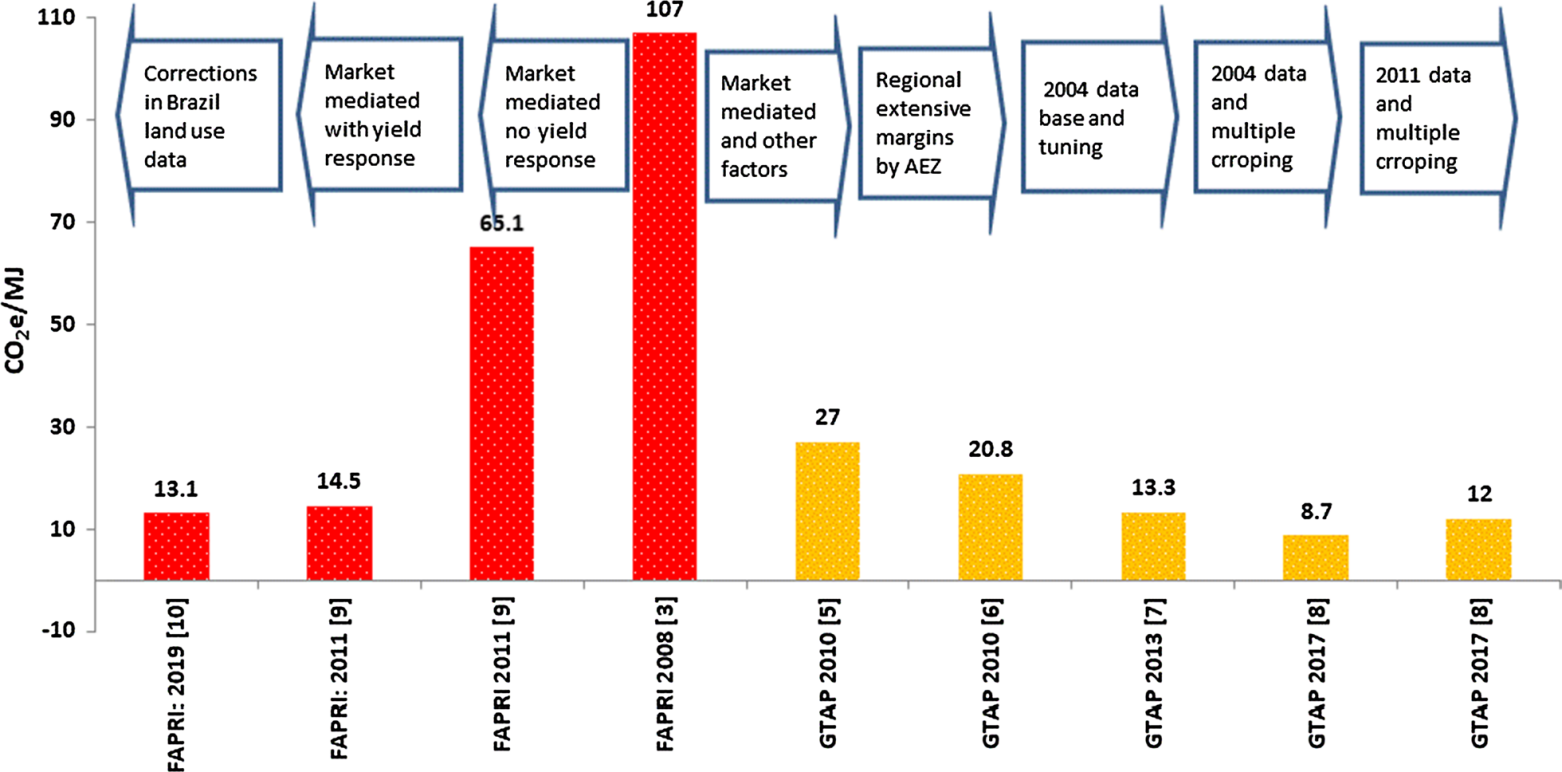
Calculated induced land use emissions (ILIC) values for US corn ethanol over time: results of FAPRI and GTAP-BIO models

The results of more recent studies that take into account actual observations and used more advanced tools show that land use changes due to biofuels have not been as large, and hence land use emissions induced by biofuels could be much smaller than early estimates. Figure 1 summarizes the outcomes of these evaluations for two different modeling frameworks that have been frequently used in these evaluations: FAPRI and GTAP-BIO [6–10]. For each model, the figure also represents the important drivers of the newer results compared to the older evaluations. Figure 1 shows that the estimated ILUC emissions for US corn ethanol declined over time for both models. The latest ILUC emissions obtained from the newer FAPRI and GTAP-BIO models are 13.1 g CO_2_e/MJ and 12 g CO_2_e/MJ, respectively. These values are roughly about one-tenth of the 100.7 g CO_2_e/MJ initially estimated by Searchinger et al. [3]. The FAPRI and GTAP-BIO are not the only models that have been used to evaluate the ILUC emissions. Besides these two models, several other economic models have been also used to assess the ILUC emissions for alternative biofuels produced across the world. Taheripour et al. [11], Khanna and Cargo [12], and Wicke et al. [13] reviewed these models, examined their differences, and compared their results. They concluded that the estimated ILUC emissions have declined over time due to model improvements, using more realistic and updated data, and tuning models to actual observations.

Regardless of these findings, still media, environmental groups, and some researchers express concerns regarding the US biofuel production and its global land use effects. In particular, more recently it has been argued that US biofuel policy is responsible for land use changes in Malaysia and Indonesia (M&I) [14, 15]. While some papers, media, and environmental groups have disseminated these concerns, no major effort has been made to address and highlight the effects of US biofuel policy on land use changes in M&I. The goal of this paper is to fill this knowledge gap.

In fact, the economic models that have been used to evaluate ILUC emissions due to biofuels commonly capture the global land use changes and their corresponding emissions by region, including the effects for M&I. However, these effects and their drivers have not been well addressed in the existing literature and have remained unclear to a large extent. In this paper, we develop analytical and numerical analyses to evaluate the extent to which production of biofuels in the US alters land use in M&I.

The analytical framework clarifies that interactions among markets for vegetable oils basically connect production of biofuels in US with land use change in M&I. It explains that the rate of substitution among vegetable oils is a key factor.

Then, as explained in “Methods” section, we modify and use a well-known CGE Model (GTAP-BIO) to numerically assess the extent to which biofuel production in US affects land use change in M&I and also the rest of the world. The numerical simulations also highlight sensitivity of the results with respect to changes in the substitution elasticity among vegetable oils. It is important to note that in the past decade several papers and research studies have developed and conducted various tests to examine the sensitivity of GTAP ILUC estimates with respect to the size of biofuel shocks, model parameters (e.g., intensive and extensive margins, trade elasticities, and regional land transformation elasticities) and emissions factors [6, 16–19]. However, none of these tests have studied the sensitivity of the results with respect to changes in the elasticity of substitution among vegetable oils. Hence, in this paper, we highlight sensitivity of land use changes and their corresponding emissions with respect to changes in this parameter, while we highlight the land use effects in M&I.

The AEZ-EF model, developed by Plevin et al. [20] and adopted by the California Air Resources Board is used to calculate these emissions [19], was used to convert the estimated land use changes to land use emissions. This model provides emissions factors for land conversion across uses and makes certain assumptions to convert land use changes to land use emissions. Among all the assumptions that this model is making, it assumes that 33% of the expansion in palm plantation in M&I occurs on the very carbon-rich peatlands of this region. Recent evidence shows that the share of palm plantation on peatlands in M&I may not be as large as 33% [21–23]. Given the uncertainty around this parameter and given that this assumption enlarges the estimated IULC values, in particular for biodiesel produced from different types of vegetable oils, we developed a sensitivity test on this assumption as well.

### Evolution in markets for vegetable oils

Global production of vegetable oils has increased rapidly over time, from about 61 Million Metric Tons (MMT) in 1990 to about 197 MMT in 2017, with an annual growth rate of 4.4%. During this time period population has increased with an annual growth rate of 1.3%. Therefore, over the past three decades global production of vegetable oils has increased more than three times faster than the population growth. Since 1990, most of the expansion in the global production of oil crops occurred in tropical countries including Brazil, Argentina, Malaysia, and Indonesia. An aggressive increase in supply of palm oil made this rapid expansion possible. In this time period, supply of palm oil (including palm kernel oil) has increased from 13 to 77 MMT, with an annual growth rate of 6.8%. Due to this extraordinary growth rate, the share of palm oil in the global supply of major vegetable oils has increased from about 21% in 1990 to 40% in 2017. Most of the expansion in supply of palm oil occurred in M&I. This region is the main producer and exporter of this product and has one of the most carbon-rich biomes on the earth [3, 20, 24, 25]. Several papers have examined the environmental consequences of this rapid change [26–31]. The main focus of this literature was the environmental damage done when peatland was converted to palm plantations. This literature also has recognized that palm plantations are not the only driver of deforestation in M&I [30, 31].

Palm oil is mainly used as a food product (about 70%) and partly used in the production processes of cosmetic products (about 25%) [25, 32]. Only a small fraction of palm oil (about 5%) was used as an energy source (including heating, electricity, and biodiesel) [32]. The share of biodiesel in global production of palm oil was less than 3% in 2016.^1^ While only a small fraction of palm oil is used for biodiesel production (mainly in the EU region), biodiesel production has been blamed for deforestation in M&I. Even more recently, it has been claimed that the US biofuel policy is responsible for deforestation in M&I [10], while the US does not use palm oil for biodiesel production and only imports a small share of the global supply of this product (e.g., about 2.2% in 2017) for food uses.

In what follows, the “Methods” section first provides a theoretical framework to explain the role of substitution among vegetable oils in linking the markets for different types of vegetable oils. In addition, the “Methods” section introduces the new changes which we made in the GTAP-BIO model to better reflect the existing links between the livestock industry, producers of oil crops, and the crushing industry that produces vegetable oils and meals (used by livestock industry) from oil crops. We do not present all components of the GTAP-BIO model, as this model is well-documented in our earlier papers. Instead, we provided proper references that present this model and its background. The “Methods” section also explains the examined experiences, including the sensitivity tests. The next section represents the “Results”, followed by a “Discussion” section. The last section makes the concluding remarks.

## Methods

### Theoretical background

The existing literature has shown that market-mediated responses and resource constraints transfer impacts of producing a particular biofuel in one region (e.g., soy biodiesel in US) to the rest of the world, and that affects global markets for agricultural products and generates land use changes across the world [5]. Among all factors that shape market-mediated responses, demand and supply elasticities^2^ play important roles. For the link between biofuel production in US and land use change in M&I, interactions among vegetable oil markets and substitution among vegetable oils play critical roles. That is because M&I are the main producers and exporters of palm oil, and the US is one of the largest producers and exporters of soybeans at the global scale. For example, in 2016, the US produced 117 MMT of soybeans, crushed 52 MMT of soybeans, and exported about 59 MMT of soybeans to other counties [32].^3^ In this analytical framework, for a given set of supplies of vegetable oils, an increase in the demand for soy biodiesel (induced by market forces or government policy) generates an additional demand for soy oil and that leads to increases in the prices of soybeans and soy oil in the US and also at the global scale, of course at different rates. Assuming some degree of substitution between palm oil and soy oil,^4^ a higher soy oil price will lead to increases in demand for palm oil. This could generate an expansion in palm plantations in M&I and lead to deforestation in this region.^5^

**Fig. 2:**
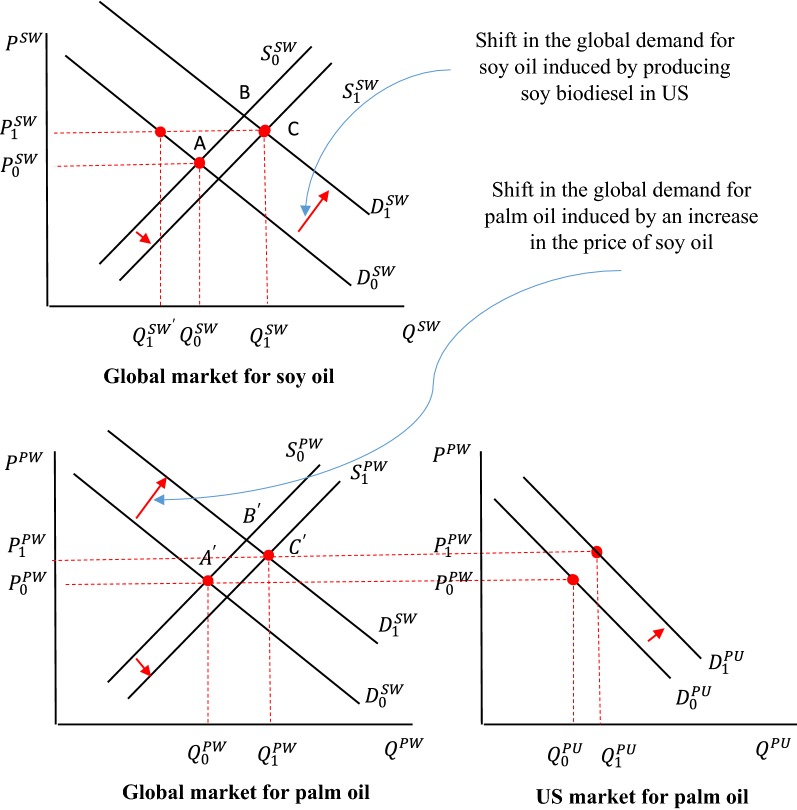
Changes in the global markets for soy and palm oil induced by producing soy biodiesel in US and its impacts on the US imports of palm oil. In this figure P represents price; Q represents quantity; superscript of PW shows global market for palm oil; superscript of PS shows global market for Soybean oil; superscript of PU stands for import demand of US for palm oil; S shows supply curve, and finally D represents demand curve

However, soy oil and palm oil are not the only vegetable oils produced and consumed across the world. The share of other vegetable oils in the global production of all major vegetable oils was about 33% in 2017, which is not a small share. Furthermore, M&I and the US are not the only players in this game. Other countries are involved in markets for oil crops and vegetable oils and produce, consume, and trade these products. Hence, in analyzing the link between production of biofuels in the US and land use changes in M&I, we should take into account the substitution among all vegetable oils at the global scale. Figure 2 depicts interactions among these markets.

The top panel of this figure represents the global market for soy oil. In this panel, the status quo equilibrium with no biodiesel production in US is shown at point $A$. At this equilibrium, the global consumption/production of soy oil would be $Q_{0}^{\text{SW}}$ at the initial price of $P_{0}^{\text{SW}}$. When the US begins converting soy oil to biodiesel, either due to market forces or a particular policy, demand for soy oil at the global scale shifts up and right from $D_{0}^{\text{SW}}$ to $D_{1}^{\text{SW}}$. Assuming no shift in the supply of soy oil, the equilibrium in this market could move to Point B. However, over time supply of soy oil may also shift right and down from $S_{0}^{\text{SW}}$ to $S_{1}^{\text{SW}}$. With these shifts in demand and supply of soy oil, market equilibrium will move to Point $C$. At this equilibrium, the price of soy oil will be $P_{1}^{\text{SW}}$ and its production will be $Q_{1}^{\text{SW}}$. At this equilibrium, the global consumption of soy oil for non-biodiesel uses will be $Q_{1}^{{{\text{SW}}{\prime }}}$ and the difference between $Q_{1}^{{{\text{SW}}{\prime }}}$ and $Q_{1}^{\text{SW}}$ shows soy oil feedstock for biodiesel production.

Changes in the soy oil market will affect the global market for palm oil as well, as presented in the bottom and left panel of Fig. 2. In this panel the status quo equilibrium with no biodiesel production in US is shown at point $A^{\prime}$. With the shift in the demand for soy oil and higher price for this product, the global demand for palm oil will shift to right and up from $D_{0}^{\text{PW}}$ and $D_{1}^{\text{PW}}$. Over time, at the global scale, the supply of palm oil will also shift to bottom and right from $S_{0}^{\text{PW}}$ and $S_{1}^{\text{PW}}$. The equilibrium point of market for palm oil will move to $C^{\prime}$ due to these changes. Due to the movement from $A^{\prime}$ to $C^{\prime},$ price of palm oil will increase from $P_{0}^{\text{PW}}$ to $P_{1}^{\text{PW}}$ and production/consumption of palm oil will increase from $Q_{0}^{\text{PW}}$ to $Q_{1}^{\text{PW}}$ at the global scale. In a CGE model, similar to our model, one can trace these changes and measure interactions between these markets. For example, one can calculate the general equilibrium cross-price elasticity of changes in the global production of palm oil (in moving from point $A^{\prime}$ to point $C^{\prime}$ in the bottom and left panel of Fig. 2) with respect to changes in the global price of soy oil (in moving from point $A$ to point $C$ in the top panel of Fig. 2) using the following formula:

$$e_{{{\text{palm}},{\text{soy}}}}^{W} = \frac{{Q_{1}^{\text{PW}} /Q_{0}^{\text{PW}} - 1}}{{P_{1}^{\text{PW}} /P_{0}^{\text{PW}} - 1}}.$$

Similarly, it is possible to calculate this measure between these markets at regional levels. For instance one can calculate the general equilibrium cross-price elasticity of palm oil production in M&I with respect to changes in the global price of soy oil.

Finally, consider the implications of changes in the global markets for soy and palm oils for the US imports of palm oil in the bottom and right panel of Fig. 2. The US status quo demand curve for imported palm oil is shown with $D_{0}^{\text{PU}}$. With this demand curve, at the status quo price of palm oil (i.e., $P_{0}^{\text{PW}}$), US imports palm oil by $Q_{0}^{\text{PU}}$. After biodiesel production, the US demand curve for imported palm oil will shift to $D_{1}^{\text{PU}}$, assuming some degrees of substitution between palm oil and soy oil. With this shift the US will import palm oil of $Q_{1}^{\text{PU}}$. The general equilibrium cross-price elasticity of changes in US palm imports with respect to its global price can be calculated using the following formula:

$$e_{{{\text{palm}},{\text{soy}}}}^{\text{US}} = \frac{{Q_{1}^{\text{PU}} /Q_{0}^{\text{PU}} - 1}}{{P_{1}^{\text{PW}} /P_{0}^{\text{PW}} - 1}}.$$

In short, Fig. 2 shows how changes in the global market for soy oil, induced by biodiesel production in US, lead to change in the global market for palm oil and that affects US demand for palm oil. These changes depend on the rate of substitution between soy and palm oils on the demand side. To develop the above analyses, we focused on the interactions between palm and soy oil. However, in the real world, in addition to these two products, other vegetable oils such as corn oil, canola oil, cotton seed oil, sunflower oil, and many more types of vegetable oils are produced and consumed across the world and their markets interact. Inclusion of these factors could significantly alter the results, as shown by our numerical analyses. Hence, in a realistic analysis one should take into account interactions among markets for all types of vegetable oils.

The CGE model that we used in this paper, aggregates all types of vegetable oils into four groups including: soy oil, palm oil, canola oil, and other vegetable oils and animal fats, and traces their changes at the global scale by country. We will use this model to examine the extent to which these markets interact at the country and global levels. The model takes into account substitution among vegetable oils by country. We examine the extent to which substitution among vegetable oils affects the interaction among vegetable oils and how that affects land use changes in M&I and their corresponding land use emissions. Using this model, we calculate the general equilibrium cross-price elasticity of changes in palm oil production in M&I with respect to changes in the price of soy oil. We show how this elasticity responds to the changes in the substitution elasticities among vegetable oils.

### Improvements in GTAP-BIO model

The latest version of the GTAP-BIO model and its background are presented in Taheripour et al. [8] and Taheripour et al. [34]. We use and improve this model to reflect the impacts of biofuel production in the US on land use changes in M&I. The improvement addresses an important aspect of the links between livestock, vegetable oil, and biofuel industries and their land use implications.

Taheripour et al. [35, 36] have shown that over time the rapid expansion in supplies of soybeans and corn have increased availability of feed products and that helped the livestock industry to produce more animal-based food products per unit of land and extend production of these food products much faster than population growth, while area of pasture land declined in recent years. This suggests that the livestock industry substituted feed for land in its production process. We modified our model to take into account this important fact.

**Fig. 3:**
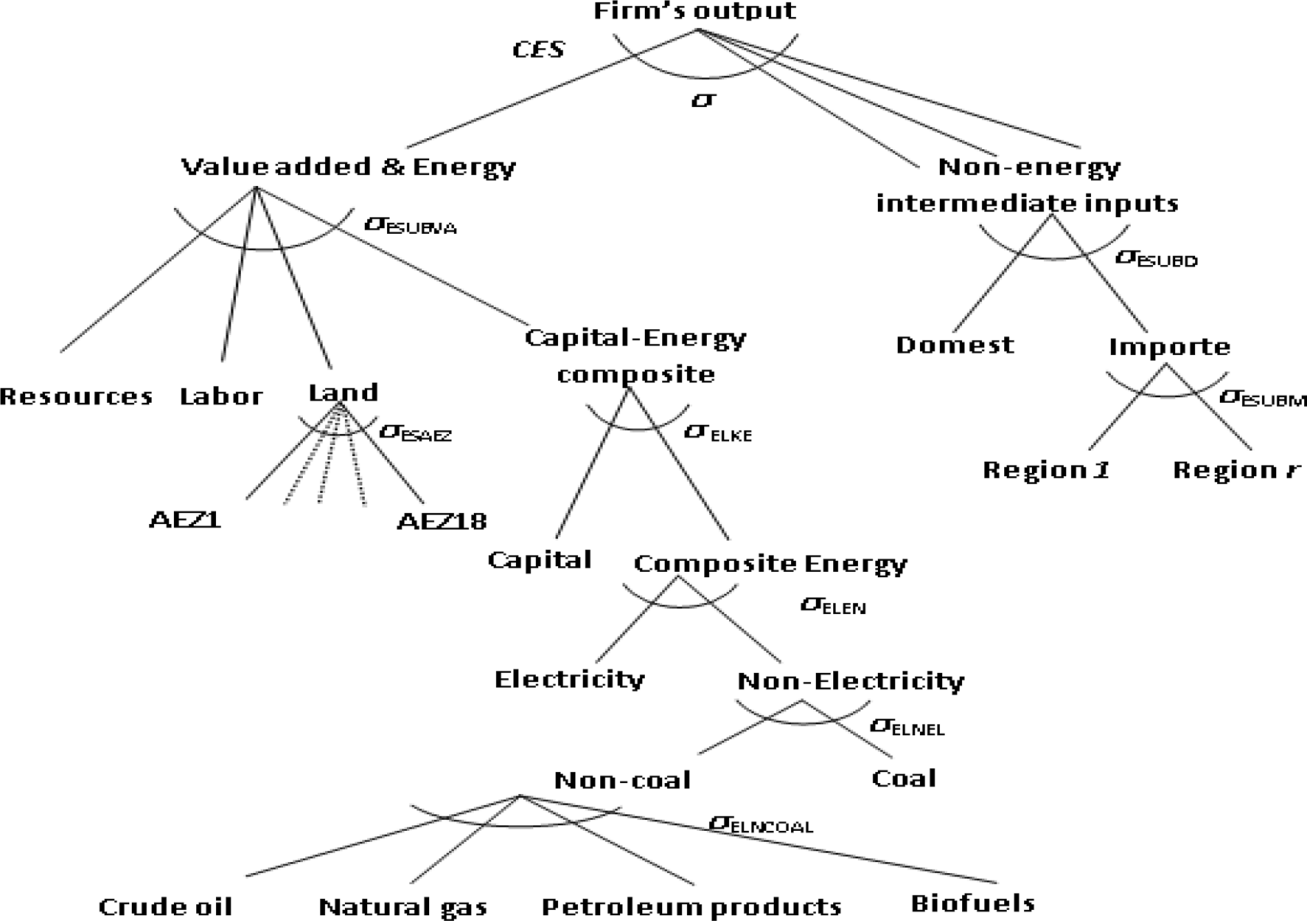
Existing nesting structure in GTAP-BIO production functions

The modification alters the nesting structure of the production functions of the GTAP-BIO model. Figure 3 represents the current structure. As shown at the top of this figure, currently this model divides all inputs into two major branches of primary (including labor, land, capital and energy) and intermediate inputs (e.g., feed items for livestock). There is no substitution at the top of this production structure. This means no substitution between feed and land. However, this structure captures some degree of substitution between land, labor, and capital, which implies some degree of land intensification in response to higher land prices (more output per unit of land) for land using sectors, including livestock.

**Fig. 4:**
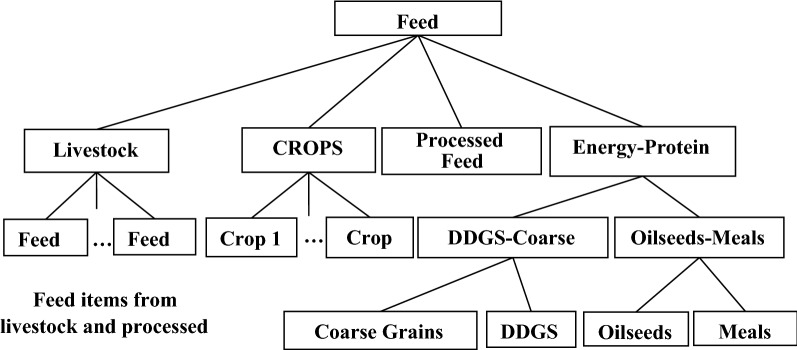
Structure of feed composite in GTAP-BIO model

On the other hand, on the branch for intermediate inputs, the current model allows substitution among feed items for the livestock industry, as shown in Fig. 4. This nesting structure allows the livestock industry to move away from more expensive feed items toward lower priced items according to the observed trends in real world (e.g., substitution between corn and DDGS or soybean meal with other protein sources).

**Fig. 5:**
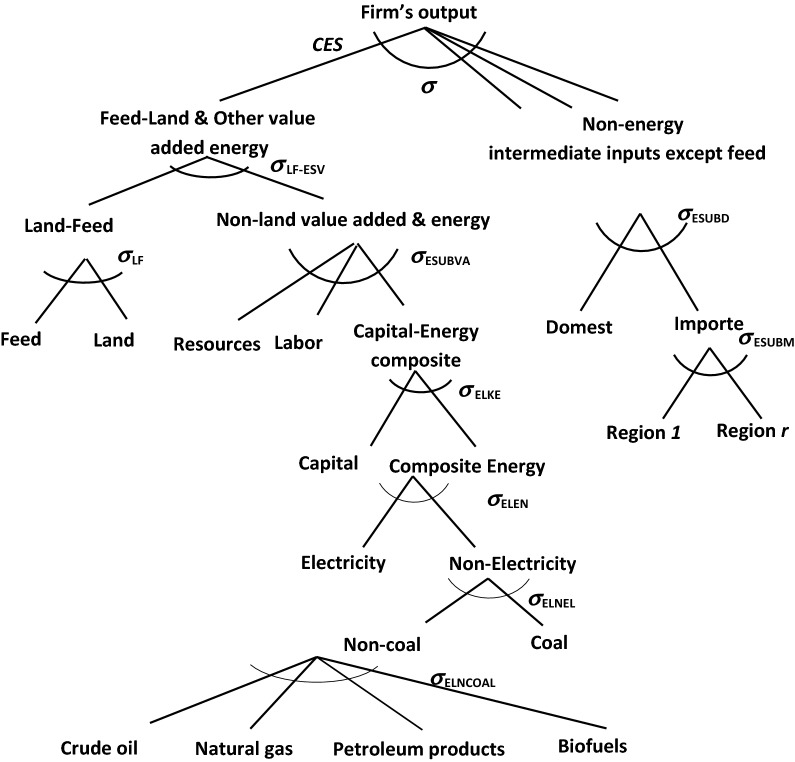
New nesting structure in GTAP-BIO production functions

In this paper, we keep the feed structure of the model as it is. However, we move the whole feed structure of the model to the first branch (the primary branch) at the top of nesting structure as shown in Fig. 5. This figure shows that in the revised model, labor, capital, and resources are bundled together, and then their mix is mingled with the mix of land and feed. Finally, the mix of primary inputs and feed is combined with other primary intermediate inputs. This arrangement takes care of the substitution between feed and land and allows the livestock industry to use more feed when the price of land goes up, and vice versa.

We introduced the substitution between land and feed demonstrated in Fig. 5 into the GTAP-BIO model reported by Taheripour et al. [8, 34]. This model uses the latest version of the GTAP-BIO database which represents the global economy in 2011. Then with this model and its database, we developed a set of simulations to tune the model to observed trends in the ratio of feed over land in recent years in the US livestock industry. We find that the implemented substitution between land and other primary inputs in the old model is also a good candidate for the substitution between land and feed. The original model allows a small degree of substitution (usually about 0.2) among primary inputs used by the livestock industry (including labor, land, and mix of energy–capital). The new model basically moves the feed item into this group and applies the original rate of substitution among them with a new nesting structure.

### Examined experiment

To examine the extent to which biofuel production in the US affects land use changes in M&I we first developed two different baseline simulations.

#### Baseline experiments

- *Corn ethanol base* Expansion in US corn ethanol by 1.07 billion gallons (BGs) from its 2011 level to 15 BGs,
- *Soy biodiesel base* Expansion in US soy biodiesel by 0.5 BGs from its 2011 level.

We refer to these cases as *corn ethanol base (CEB)*, and *soy biodiesel base (SBB)*. In these simulations, we use the standard GTAP-BIO parameters including a set of regional parameters that govern substitution among vegetable oils at the global scale. These parameters have been used by the California Air Resources Board (CARB) in evaluating land use emissions due to biofuels.

Table 1 represents these parameters. As shown in this table, the regional substitutions are relatively large except for US, Brazil, and South and Central America. These exceptional regions are big soybean producers. They basically consume soybean oils from their own produced soybeans and commonly import limited amounts of other types of vegetable oils. In other regions, there is a combination of production, trade, and consumption of vegetable oils, and we use relatively large substitution elasticities of 5 and 10. In particular, M&I, China, and India which produce/consume large quantities of palm oil in combination with other types of vegetable oils are assigned a large substitution elasticity of 10.

**Table 1:** Regional substitution elasticities among vegetable oils in GTAP-BIO model

Region	Elasticity values	Region	Elasticity values
US	0.5	M&I	10.0
EU	5.0	Rest of South East Asia	5.0
Brazil	0.5	Rest of South Asia	5.0
Canada	5.0	Russia	5.0
Japan	5.0	Central and Eastern Europe	5.0
China	10.0	Other Europe	5.0
India	10.0	Middle East and North Africa	5.0
Central America	0.5	Sub-Saharan Africa	5.0
South America	0.5	Oceania	5.0
East Asia	5.0		

These values are taken from California Air Resources Board [19]

To examine the sensitivity of induced land use changes with respect to changes in the regional substitution elasticities among vegetable oils, we examined several sets of experiments. The first set examines global land use changes and the second one concentrates more on land use changes in M&I. In the first set, we examined the following cases for each biofuel including ethanol and biodiesel.

#### First set of sensitivity tests

- Test 1: repeat the baseline cases with an increase in the regional substitution elasticities among vegetable oils by 25%,
- Test 2: repeat the baseline cases with an reduction in the regional substitution elasticities among vegetable oils by − 25%,
- Test 3: repeat the baseline cases using a global uniform low substitution elasticity of 0.5 among vegetable oils,
- Test 4: repeat the baseline cases using a global uniform substitution elasticity of 1.0 among vegetable oils,
- Test 5: repeat the baseline cases using a global uniform substitution elasticity of 3.0 among vegetable oils,
- Test 6: repeat the baseline cases using a global uniform substitution elasticity of 5.0 among vegetable oils.

The ± 25% tests show a range that commonly is used for a sensitivity test in the GTAP selectivity tests. However, one can conduct this test for other ranges as well. We represent each of these cases with the name of examined biofuel and the test number. For example, *Corn Ethanol Test 1 (CET1)* or *Soy Biodiesel Test 1 (SBT1)*.

#### Second set of sensitivity tests

The second set of sensitivity tests concentrates more on the land use changes in M&I in response to the changes in the substitution elasticity among vegetable oils only in the US, while we use the original substitutions elasticities for other countries and regions. As explained in the “Results” section, producing soy biodiesel leads to more land use changes in M&I. For this reason, in this set of tests we concentrate on production of soy biodiesel. As mentioned before, the base value for the substitution among vegetable oils is about 0.5 for the US. In addition to the base value, in this set of experiments we assign the values of 0.25, 0.75, 1, 2, 5 and 10 to this parameter and repeat the base case simulation for soy biodiesel. We evaluate these tests under two different alternative scenarios on modeling vegetable oils and oil crops.

In the first scenario, we allow all types of vegetable oils and oil crops to respond to the expansion in biofuels, as happens in real world. We refer to the simulations of this scenario as “*Unrestricted”* experiments. In the second scenario, we alter the model setup to only take into account palm oil and soy oil and drop all other vegetable oils and oil crops. We refer to this set of simulations as “*Restricted”* experiments. The “restricted” experiments follow the literature that only takes into account interactions between palm oil and soy oil and ignores other vegetable oils [10]. Table 2 summarizes the second set of sensitivity experiments and their corresponding names.

**Table 2:** Experiments included in the second set of sensitivity test for an expansion in US soy biodiesel by 0.5 billion gallons

Size of substitution elasticity among vegetable oils	Unrestricted experiments: all vegetable oils and oil crops are included	Restricted experiments: only palm and soybean oils and crops are included
0.25	SBUT1	SBRT1
0.50	SBUT2 (or SBB)	SBRT2
0.75	SBUT3	SBRT3
1.0	SBUT4	SBRT4
2.0	SBUT5	SBRT5
5.0	SBUT6	SBRT6
10.0	SBUT7	SBRT7

## Results

### Base cases results

Table 3 represents the land use changes and their corresponding emissions for corn ethanol and soy biodiesel produced in US. The expansion in corn ethanol (by about 1.07 BGs) increases global area of cropland by about 68.3 thousand hectares. The share of M&I in this land requirement for ethanol production is about 3.5%, about 2.4 thousand hectares. The results suggest that an increase in US corn ethanol by 1 BGs gallons would increase area of cropland in M&I by 2.24 thousand hectares. Therefore, according to the simulation results, producing 15 BGs of corn ethanol in US could increase area of cropland in M&I by about 33.5 thousand hectares.^6^

**Table 3:** Land use changes and their corresponding emissions for corn ethanol and biodiesel produced in US

Biofuel	Land type	US	EU27	Brazil	M&I	Other	Total
Corn ethanol	Forest area (ha)	1808	− 1232	− 4192	− 2312	− 19,366	− 25,294
	Pasture area (ha)	− 6048	− 1768	− 7504	− 62	− 27,774	− 43,156
	Cropland area in (ha)	4240	2928	11,712	2392	47,065	68,337
	Share in cropland (%)	6.2	4.3	17.1	3.5	68.9	100.0
	Land use emissions	12.3 g CO_2_e MJ^−1^ or 1001 g CO_2_e EGe^−1^					
	Share in emissions (%)	11.1	2.8	21.7	10.9	53.5	100.0
Soy biodiesel	Forest area (ha)	512	− 176	− 432	− 5892	6161	173
	Pasture area (ha)	− 2688	− 1032	− 5296	− 596	− 27,886	− 37,498
	Cropland area (ha)	2176	1192	5724	6504	21,732	37,328
	Share in cropland (%)	5.8	3.2	15.3	17.4	58.2	100.0
	Land use emissions	17.5 g CO_2_e MJ^−1^ or 1424 g CO_2_e EGe^−1^					
	Share in emissions (%)	8.5	1.0	6.6	78.0	5.9	100.0

The AEZ-EF model developed by Plevin et al. [20] is used to calculate emissions

Table 3 shows that the expansion in soy biodiesel (by 0.5 billion gallons) increases global area of cropland by about 37.3 thousand hectares. The share of M&I in this land requirement is 17.4%, about 6.5 thousand hectares. This suggests that an increase in US soy biodiesel by 1 BGs gallons could extend area of cropland in M&I by 13 thousand hectares. This result indicates that producing 2 BGs of soy biodiesel in US could increase area of cropland in M&I by about 26 thousand hectares.^7^

Therefore, producing 15 BGs of corn ethanol and 2 BGs gallons of soy biodiesel together could potentially increase area of cropland in M&I by 59.6 thousand hectares.^8^ This figure is really negligible compared to the scale and magnitude of land conversion in M&I, where area of cropland has increased by 11.7 million hectares between 2000 and 2016. These results suggest that less than 0.5% of the cropland expansion in M&I for the time period of 2000–2016 could be assigned to the expansion in biofuels in the US.

The estimated induced land use emissions for US corn ethanol is about 12.3 g CO_2_e MJ^−1^. As shown in Table 3, the share of M&I in land use emissions for this biofuel is about 10.9%, more than three times higher than the land share. That is because the land use emission factors for M&I are significantly larger than the emissions factors of other countries.

The estimated induced land use emissions for US soy biodiesel is about 17.5 g CO_2_e MJ^−1^. As shown in Table 3, the share of M&I in land use emissions for this biofuel is about 78%, about 4.5 times higher than the land share.^9^ Two factors explain this extremely large share. The large emission factors of M&I partially explain this observation. The low meal content of oil palm compared with the meal content of other oil crops is another factor that also partially explains the high share of M&I in the estimated land use emissions for US soy biodiesel. As explained earlier, an expansion in soy biodiesel increases the demand and eventually production of oil crops in US and other regions. In those regions which produce high meal-content oil crops (e.g., soybeans), the livestock industry uses the additional meals, and that reduces their demand for pasture land. In these regions land conversion falls on pasture land, as an example see Taheripour et al. [36]. In M&I, which produces oil palm with low meal content, the land conversion falls mainly on forest and peatland with extremely high emission factors. This analysis confirms that the substitution among vegetable oils and low meal content of oil palm play important roles in land use emissions induced by US soy biodiesel.

The AEZ-EF model [20] which converts land use changes to land use emissions assumes that 33% of the expansion in oil palm plantations in M&I falls on peatland with very high rate of emissions. More recent evidence indicates that this assumption is not consistent with recent observations and the share of palm plantation on peatland is less than 33% [21–23]. To examine the extent to which this assumption affects the results, we estimated the land use emissions for the base cases with 20% and 10% shares of palm plantation on peatland as well. For corn ethanol, the size of ILUC emissions drops from 12.3 g CO_2_e MJ^−1^ with the 33% assumption to 12.11 g CO_2_e MJ^−1^ and 11.96 g CO_2_e MJ^−1^ with 20% and 10% assumptions, respectively. Therefore, the results indicate that the size of ILUC emissions value for the case of corn ethanol is not very sensitive to the share of palm on peatland. However, the size of ILUC emissions value for the case of soy biodiesel is very sensitive to the share of palm on peatland. For soy biodiesel the size of ILUC emissions value drops from 17.5 g CO_2_e MJ^−1^ with the 33% assumption to 14 g CO_2_e MJ^−1^ and 10.4 g CO_2_e MJ^−1^ with 20% and 10% assumptions, respectively.

### First set of sensitivity tests results

The results of this set of sensitivity tests are included in Table 4 for the cases of US corn ethanol. This table clearly shows that the land use and land use emissions for corn ethanol do not vary significantly with changes in the substitution elasticity among vegetable oils. Table 5 shows the results for the case of US soybean biodiesel. From this table one can infer that:

**Table 4:** Land use changes and their corresponding emissions for the first set of examined sensitivity test for US corn ethanol (land areas are in hectare)

Base case	Land type	USA	UE27	Brazil	Mala-Indo	Other	Total
	Cropland	4240	2928	11,712	2392	47,065	68,337
	Forest	1808	− 1232	− 4192	− 2312	− 19,366	− 25,294
	Pasture	− 6048	− 1768	− 7504	− 62	− 27,774	− 43,156
	Land use emissions	12.31 g CO_2_ e MJ^−1^ or 1001 g CO_2_e EGe^−1^					
Test 1	Land type	USA	UE27	Brazil	Mala-Indo	Other	Total
	Cropland	4240	2928	11,668	2392	46,996	68,224
	Forest	1808	− 1200	− 4176	− 2320	− 19,393	− 25,281
	Pasture	− 6048	− 1760	− 7504	− 62	− 27,731	− 43,105
	Land use emissions	12.31 g CO_2_ e MJ^−1^ or 1001 g CO_2_e EGe^−1^					
Test 2	Land type	USA	UE27	Brazil	Mala-Indo	Other	Total
	Cropland	4240	2920	11,760	2360	47,103	68,383
	Forest	1808	− 1232	− 4224	− 2292	− 19,359	− 25,299
	Pasture	− 6064	− 1768	− 7552	− 60	− 27,822	− 43,267
	Land use emissions	12.29 g CO_2_ e MJ^−1^ or 999 g CO_2_e EGe^−1^					
Test 3	Land type	USA	UE27	Brazil	Mala-Indo	Other	Total
	Cropland	4272	2936	11,960	2144	47,292	68,604
	Forest	1808	− 1232	− 4304	− 2084	− 19,432	− 25,244
	Pasture	− 6080	− 1772	− 7680	− 44	− 27,979	− 43,555
	Land use emissions	12.02 g CO_2_ e MJ^−1^ or 978 g CO_2_e EGe^−1^					
Test 4	Land type	USA	UE27	Brazil	Mala-Indo	Other	Total
	Cropland	4240	2928	11,884	2176	47,165	68,393
	Forest	1808	− 1232	− 4272	− 2132	− 19,389	− 25,217
	Pasture	− 6064	− 1768	− 7616	− 47	− 27,924	− 43,419
	Land use emissions	12.05 g CO_2_ e MJ^−1^ or 980 g CO_2_e EGe^−1^					
Test 5	Land type	USA	UE27	Brazil	Mala-Indo	Other	Total
	Cropland	4192	2928	11,668	2264	46,866	67,918
	Forest	1808	− 1216	− 4192	− 2212	− 19,345	− 25,157
	Pasture	− 6032	− 1760	− 7488	− 54	− 27,537	− 42,871
	Land use emissions	12.07 g CO_2_ e MJ^−1^ or 981 g CO_2_e EGe^−1^					
Test 6	Land type	USA	UE27	Brazil	Mala-Indo	Other	Total
	Cropland	4192	2904	11,544	2264	46,655	67,559
	Forest	1808	− 1184	− 4160	− 2236	− 19,369	− 25,141
	Pasture	− 6016	− 1748	− 7408	− 55	− 27,287	− 42,514
	Land use emissions	12.02 g CO_2_ e MJ^−1^ or 977 g CO_2_e EGe^−1^

**Table 5:** Land use changes and their corresponding emissions for the first set of examined sensitivity test for US soy biodiesel (land areas are in hectare)

Base case	Land type	USA	UE27	Brazil	Mala-Indo	Other	Total
	Cropland	2176	1192	5724	6504	21,732	37,328
	Forest	512	− 176	− 432	− 5892	6161	173
	Pasture	− 2688	− 1032	− 5296	− 596	− 27,886	− 37,498
	Land use emissions	17.51 g CO_2_ e MJ^−1^ or 1424 g CO_2_e EGe^−1^					
Test 1	Land type	USA	UE27	Brazil	Mala-Indo	Other	Total
	Cropland	2112	1216	5176	6880	21,069	36,453
	Forest	496	− 176	− 240	− 6236	6189	33
	Pasture	− 2624	− 1040	− 4912	− 622	− 27,375	− 36,573
	Land use emissions	17.72 g CO_2_ e MJ^−1^ or 1441 g CO_2_e EGe^−1^					
Test 2	Land type	USA	UE27	Brazil	Mala-Indo	Other	Total
	Cropland	2240	1184	6392	5968	22,548	38,332
	Forest	528	− 176	− 608	− 5408	5959	295
	Pasture	− 2768	− 1028	− 5712	− 558	− 28,528	− 38,594
	Land use emissions	17.14 g CO_2_ e MJ^−1^ or 1394 g CO_2_e EGe^−1^					
Test 3	Land type	USA	UE27	Brazil	Mala-Indo	Other	Total
	Cropland	2384	1104	9136	1752	24,612	38,988
	Forest	560	− 176	− 1488	− 1492	5290	2694
	Pasture	− 2864	− 960	− 7648	− 255	− 29,920	− 41,647
	Land use emissions	10.16 g CO_2_ e MJ^−1^ or 826 g CO_2_e EGe^−1^					
Test 4	Land type	USA	UE27	Brazil	Mala-Indo	Other	Total
	Cropland	2256	1096	8008	2912	23,126	37,398
	Forest	528	− 144	− 1120	− 2584	5631	2311
	Pasture	− 2768	− 932	− 6864	− 338	− 28,858	− 39,761
	Land use emissions	11.99 g CO_2_ e MJ^−1^ or 975 g CO_2_e EGe^−1^					
Test 5	Land type	USA	UE27	Brazil	Mala-Indo	Other	Total
	Cropland	1872	1000	5032	5256	19,078	32,238
	Forest	384	− 144	− 288	− 4744	6346	1554
	Pasture	− 2192	− 876	− 4752	− 504	− 25,396	− 33,720
	Land use emissions	13.98 g CO_2_ e MJ^−1^ or 1137 g CO_2_e EGe^−1^					
Test 6	Land type	USA	UE27	Brazil	Mala-Indo	Other	Total
	Cropland	1600	920	3300	6144	16,417	28,381
	Forest	272	− 128	176	− 5556	6705	1469
	Pasture	− 1872	− 832	− 3472	− 565	− 23,340	− 30,081
	Land use emissions	14.90 g CO_2_ e MJ^−1^ or 1211 g CO_2_e EGe^−1^

- A 25% increase in the regional substitution elasticities among vegetable oils generates more deforestation in EU27, Brazil, and M&I and that barely increases the estimate of land use emissions from 17.5 to 17.7 g CO_2_e MJ^−1^.
- A 25% reduction in the regional substitution elasticities among vegetable oils generates less deforestation in EU27, Brazil, and M&I and that barely reduces the estimate of land use emissions from 17.5 to 17.3 g CO_2_e MJ^−1^.
- Applying a uniform and small substitution elasticity of 0.5 among all vegetable oils across the world reduces induced land use changes for soybean biodiesel. That reduces land use emissions from 17.5 g CO_2_e MJ^−1^ for the base case to 10.2 g CO_2_ e MJ^−1^. The existing evidence does not confirm such a low level of substitution elasticity in many regions across the world.^10^
- The results for tests SBT4, SBT5, and SBT6 show that land use emissions grow as we apply larger uniform substitution elasticities, and that is basically because with more substitution among vegetable oils more deforestation occurs in M&I. The results of these tests indicate that as we apply larger substitution elasticities among vegetable oils, the induced land use emissions converge towards the base cases results.

### Second set of sensitivity tests results

**Fig. 6:**
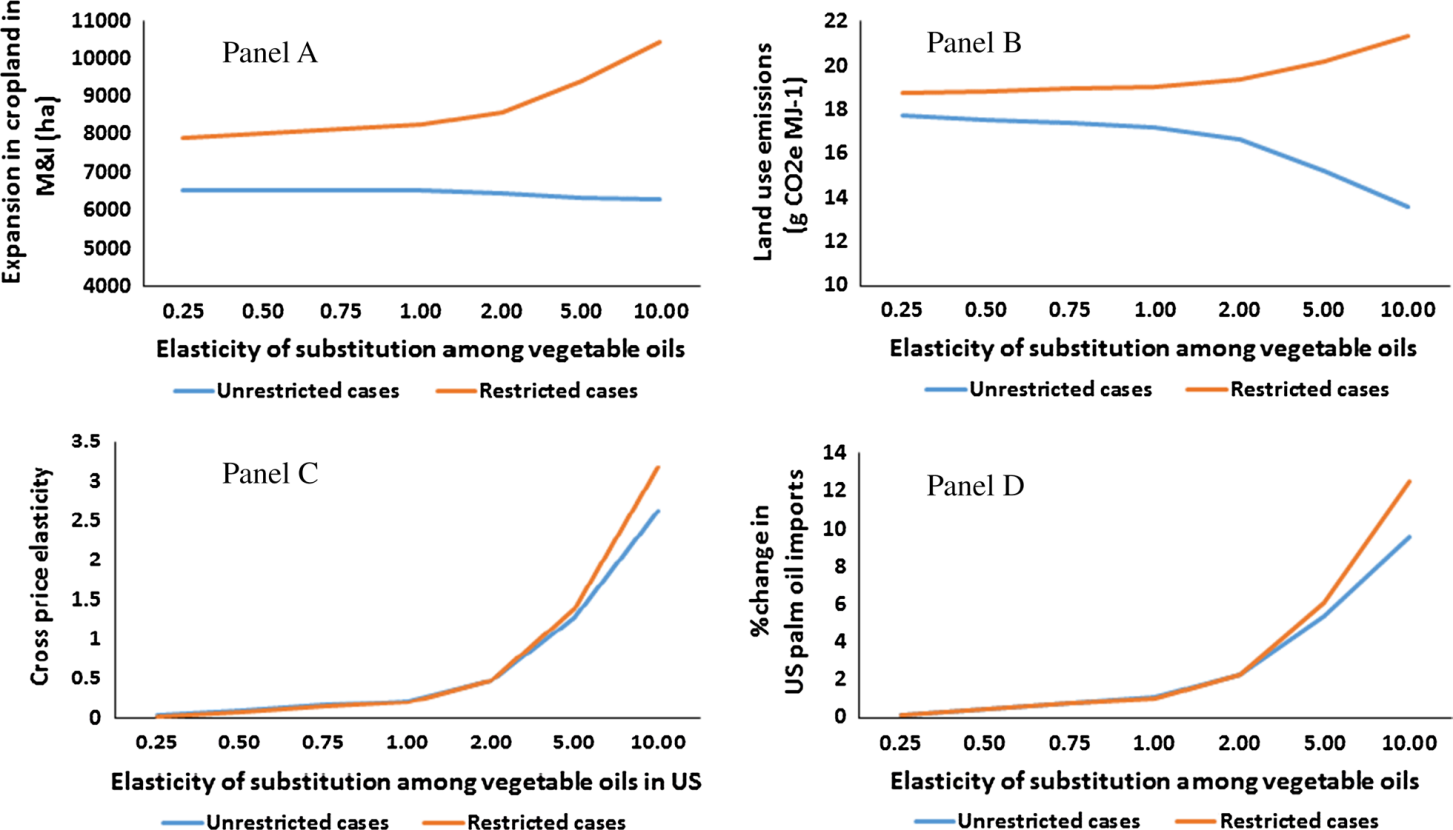
Implications of an increase in US soy biodiesel by 0.5 billion gallons on: (1) area of cropland in M&I (**A**); global land use emissions (**B**); (2) cross-price elasticity of palm oil with respect to soy oil price (**C**) and; imposts of US palm oil (**D**) under alternative substitution elasticities among vegetable oils. Unrestricted means all oil markets are included. Restricted means only soy and palm oils are included

Figure 6 summarizes the results of this set of experiments in four panels. For the unrestricted cases, panel A of this figure shows increases in the area of cropland in M&I due the expansion in US soy biodiesel by 0.5 BGs for all of the examined substitution elasticities for US. As shown in this figure, with the lowest examined substitution elasticity (i.e., 0.25) area of cropland in M&I increases by 6.5 thousand hectares and then it gradually and slightly drops down to 6.3 thousand hectares for the highest examined substitution (i.e., 10) when markets for all types of vegetable oils and animal fats are included in the model, see the blue line in panel A of Fig. 6. This suggests that with a larger substitution elasticity among all types of vegetable oils and animal fats in the US, less land use changes occur in M&I. That is due to the fact that a larger substitution elasticity diverts a larger portion of the additional demand for soy oil to non-palm vegetable oils and animal fats that are produced either in the US or regions other than M&I.

The reverse can be observed for the *restricted* case when we only take into account soy and palm oils and ignore all other types of vegetable oils and animal fats. In the *restricted* cases, with the lowest substitution elasticity (i.e., 0.25), the area of cropland in M&I increases by 7.9 thousand hectares and then it grows relatively fast to 104 thousand hectares with the highest examined substitution elasticity (i.e., 10), see the orange line in panel A of Fig. 6. These results clearly indicate that those analyses that limit their modeling framework to only palm and soy oil and ignore other types of vegetable oils and fats provide misleading information and exaggerate about the land use implications of the US biofuels for M&I. As an example of this type of analysis see Santeramo and Searle [15].

Panel B of Fig. 6 shows the land use emissions for US soy biodiesel for all of the examined substitution elasticities for the *restricted* and *unrestricted* cases. This panel shows that for the *unrestricted* cases, the magnitude of land use emissions drops as we use larger substitution elasticities in US, see the blue line in this panel. For the restricted cases one can see the reverse direction: the higher the substitution elasticity the higher the land use emissions, see the orange line in this panel.

In our theoretical analyses, we explained how the substation elasticity among vegetable oils links the oil markets—the larger the substitution in oil consumption the stronger the link between oil markets. The general equilibrium cross-price elasticities measure this link. Using the results of the second set of sensitivity tests, we measured the cross-price elasticity of palm oil with respect to the price of soybean, $e_{{{\text{palm}},{\text{soy}}}}^{\text{W}} = \frac{{Q_{1}^{\text{PW}} /Q_{0}^{\text{PW}} - 1}}{{P_{1}^{\text{PW}} /P_{0}^{\text{PW}} - 1}}$, for the restricted and unrestricted cases. Panel C of Fig. 6 represents the relationship between these cross elasticities and the examined substitution elasticities for the US economy. This panel clearly shows that:

- The sign of cross-price elasticity is positive, which suggests an increase in soy oil price leads to an increase in production/consumption of palm oil,
- The magnitude of cross-price elasticity remains under 0.5 for all substitution elasticities below 2.
- The *unrestricted* and *restricted* cases provide identical cross-price elasticities for low substitution elasticities. At higher substitution rates, the restricted cases provide larger cross-price elasticities.

Finally, panel D of Fig. 6 represents changes in US palm oil imports due to the expansion in soy biodiesel by 0.5 BGs. This panel indicates that as the size of substitution among vegetable oils increases, US imports more palm oil under the unrestricted and restricted scenarios. However, the percent changes in palm oil imports remain limited, even under large substitution elasticities. Given that the size of US palm imports is limited (less than 2% of the palm produced across the world), these results confirm that the implications of producing soy biodiesel for the palm oil market will be very limited.

## Discussion

Following the rapid expansion in biofuel production across the world, numerous studies have examined the land use effects of alternative biofuel pathways produced across the world. While the existing literature on this topic clearly confirms that the early published papers in this area exaggerated these effects, still media, environmental groups, and some researchers express concerns about these effects and even sometimes argue that the US biofuel policy is responsible for deforestation in M&I. In response to these arguments, following a short literature review that highlights the relevant topics and issues, we developed analytical and numerical analyses to study the extent to which production of biofuels in the US would affect land use in M&I. The analytical analyses make it clear that market-mediated responses may generate land use change in M&I due to biofuel production in the US. These analyses highlight the role of substitution among vegetable oils for the case of biodiesel. We discussed the role of this elasticity of substitution in linking the global markets for vegetable oils and the way that these links and market-mediated responses shift the land use effect of producing soy biodiesel in the US to M&I.

To numerically quantify these effects, we modified and used a well-known CGE model, GTAP-BIO. To be more specific, we examined the effects of US corn ethanol and soy biodiesel production. Our numerical analyses first developed two simulations, one for ethanol and one for soy biodiesel, with the model baseline parameters, including a set of regional substitution elasticities among vegetable oils which has been adopted by the CARB. These regional elasticities are large (larger than 5) except for those countries that basically produce and use soybean oils. To test the sensitivity of our results with respect to changes in these elasticities we examined several experiments. Note that several papers and research reports have tested sensitivity of GTAP-BIO results with respect to the key parameters that affect ILUC emissions. However, they did not perform this test for the elasticity of substitution among vegetable oils. Hence in this paper, we developed sensitivity analyses on this particular parameter.

According to the simulation results obtained for the base case scenarios for corn ethanol and soy biodiesel, we concluded that producing 15 BGs of corn ethanol and 2 BGs gallons of soy biodiesel together could potentially increase area of cropland in M&I by 59.6 thousand hectares. That is less than 0.5% of the cropland expansion in M&I for the time period of 2000–2016, when biofuel production increased in the US.

Our results show that 10.9% of the estimated ILUC emissions value for corn ethanol (12.3 g CO_2_e MJ^−1^) is due to land use changes in M&I in the base case scenario. These results do not change significantly with the changes in the substitution elasticity among vegetable oils. These results show that production of corn ethanol induces some small land use changes in M&I, but these changes are not sensitive to the changes in the markets for vegetable oils.

We show that 78% of the estimated ILUC emissions value for soy biodiesel (17.5 g CO_2_e MJ^−1^) is due to large land use emissions factors in M&I in the base case scenario. However, unlike the case of corn ethanol, these results are sensitive with respect to the changes in the regional rates of substitution among vegetable oils. Our sensitivity tests indicate that other factors being equal:

1. The ILUC emissions value for soy biodiesel does not change significantly with ± 25% change in the base case regional substitution elasticities. This is due to the fact that the original substitution elasticities used in the model are large (larger than 5) for the regions that import and use a mix of different vegetable oils. A large substitution elasticity (such as 5 or 10) is still large after a 25% change (in either direction).
2. When we used a small substitution rate (i.e., 0.5) uniformly all across the world, the estimated ILUC emissions value for soy biodiesel declined from 17.5 g CO_2_e MJ^−1^ to 10.16 g CO_2_e MJ^−1^.
3. When we gradually increased the implemented substitution rate from 0.5 to 5, the estimated ILUC emissions values for soy biodiesel followed an increasing trend with a diminishing rate from 10.16 g CO_2_e MJ^−1^ to 14.9 g CO_2_e MJ^−1^,
4. The results of these tests indicate that as we apply larger substitution elasticities among vegetable oils, the estimated ILUC emissions value for soy biodiesel converges towards the base case results.

For the proportion of oil palm plantations on peatland in M&I, our results show that the size of ILUC emissions value for soy biodiesel is very sensitive to this share. For soy biodiesel the size of ILUC emissions value drops from 17.5 g CO_2_e MJ^−1^ with the 33% assumption to 14 g CO_2_e MJ^−1^ and 10.4 g CO_2_e MJ^−1^ with 20% and 10% assumptions, respectively. Since the AEZ-EF model assumes 33% for the share of oil palm on peatland and the new research shows that the proportion of oil palm on peatland, is significantly less than 33%, we can conclude that the AEZ-EF model overestimates the estimated ILUC values for soy biodiesel.

Finally, our results confirm that with a larger substitution elasticity among all types of vegetable oils and animal fats in US, less land use changes occur in M&I. That is due to the fact that a larger substitution elasticity among vegetable oils in US, diverts a larger portion of the additional demand for soy oil to non-palm vegetable oils and animal fats that are produced either in the US or regions other than M&I. Our analyses clearly indicate that those analyses that limit their modeling framework to only palm and soy oils and ignore other types of vegetable oils and fats provide misleading information and exaggerate the land use implications of the US biofuels for M&I.

## Conclusions

The main conclusions of this paper are:

1. Production of biofuels in the US generates some land use effects in M&I due to market-mediated responses, in particular through the links between markets for vegetable oils. These effects are minor compared with the magnitude of the overall observed land use changes in M&I. However, because of the high carbon intensity of the peatland the emissions fraction of M&I is larger, in particular for soy biodiesel.
2. The GTAP-BIO model implemented a set of regional substitution elasticities among vegetable oils that, other factors being equal, provides the largest possible ILUC emissions value for soy biodiesel.
3. With a larger substitution elasticity among all types of vegetable oils and animal fats in the US, less land use changes occur in M&I. That is due to the fact that a larger substitution elasticity among vegetable oils in the US, diverts a larger portion of the additional demand for soy oil to non-palm vegetable oils and animal fats that are produced either in the US or regions other than M&I.
4. Those analyses that limit their modeling framework to only palm and soy oils and ignore other types of vegetable oils and fats provide misleading information and exaggerate the land use implications of the US biofuels for M&I.

## Data Availability

The GTAP data base is publically available on the GTAP web site at http://www.gtap.org.

## Abbreviations

GTAP-BIO: Global Trade Analysis Project with Biofuels
ILUC: induced land use change
BG: billion gallons
GDP: gross domestic product
EU: European Union
MMT: million metric tons
DDGS: distillers dried grains with solubles
US: United States
CGE: Computable General Equilibrium
M&I: Malaysia and Indonesia
AEZ-EF: agro-ecological zone-emission factor
CEB: corn ethanol base
SBB: soy biodiesel base
CET1: Corn Ethanol Test 1
SBT1: Soy Biodiesel Test 1
FAPRI: Food and Agricultural Policy Research Institute

## Footnotes

1 This share is calculated based on the share of palm biodiesel in the global production of biodiesel in 2016 obtained from Kim et al. [33] and global production of palm oil in 2016 obtained from the oilseeds: World Markets and Trade report [32].

2 To develop numerical analyses one needs to empirically determine these elasticities.

3 The discrepancy between production and consumption (exported and crushed soybeans) is due to changes in the stock and some imports.

4 The rate of substitution between palm oil and soybean oil represents the market parity for these products.

5 Production of corn ethanol also affects land use changes in M&I through the markets for vegetable oils as corn and soy are two major crops in the US. Producing more corn for ethanol could reduce production of soybeans and that could alter the markets for vegetable oils as well.

6 Here, we made a simple extrapolation since the size of ethanol shock and its regional land requirement follow a fairly linear relationship in GTAP.

7 The previous comment is valid for the case of soybean biodiesel as well.

8 In this calculation, the 15 BGs of corn ethanol and 2 BGs of soy biodiesel represent the RFS-mandated levels of conventional ethanol and biodiesel for 2017. However, the RFS has not specified the biodiesel feedstock.

9 As shown in Table 3, for the case of soy diesel, the share M&I in emissions is 78% and its share in cropland expansion is 17.4%. Therefore the share of emissions is 4.5 times of the share of cropland.

10 Only a few efforts have tried to quantify the rate of substitution among vegetable oils. These efforts tried to quantify this substitution elasticity by estimating the own and cross price elasticities of demand for vegetable oils mainly for the US and EU economies [37–40]. While these papers provide a mix of evidence for the rate of substitution among vegetable oils for these economies, they usually refer to the high correlation rates between the prices of these products to argue that the rate of substitution among vegetable oils is not small.
